# FoxP3^+^ and IL-17^+^ cells are correlated with improved prognosis in cervical adenocarcinoma

**DOI:** 10.1007/s00262-015-1678-4

**Published:** 2015-03-21

**Authors:** Simone Punt, Marjolein E. van Vliet, Vivian M. Spaans, Cornelis D. de Kroon, Gert Jan Fleuren, Arko Gorter, Ekaterina S. Jordanova

**Affiliations:** 1 Department of Pathology, Leiden University Medical Center, Leiden, The Netherlands; 2 Department of Gynaecology, Leiden University Medical Center, Leiden, The Netherlands; 3 Center for Gynecological Oncology Amsterdam, VUMC, Amsterdam, The Netherlands

**Keywords:** Uterine cervical cancer, Adenocarcinoma, Tumor microenvironment, Treg, Th17, IL-17

## Abstract

Cervical adenocarcinoma comprises approximately 15 % of cervical cancer cases. This histological subtype has different characteristics than cervical squamous cell carcinoma, which may influence disease progression. To study whether the infiltration of T cell subpopulations was correlated with cervical adenocarcinoma patient survival, similar to squamous cell carcinoma, the tumor-infiltrating T cells, Tregs, Th17 cells and IL-17^+^ cell frequencies were analyzed in a cohort of cervical adenocarcinoma patients (*n* = 67). Intraepithelial, stromal and total cell frequencies were scored using triple immunofluorescence. The majority of Tregs were present in the tumor stroma, while other T cells and IL-17^+^ cells infiltrated the tumor epithelium three times more frequently. A high total number of Tregs were significantly correlated with improved disease-specific and disease-free survival (*p* = 0.010, *p* = 0.007). Within the tumor epithelium, a high T cell frequency was significantly correlated with improved disease-free survival (*p* = 0.034). In particular, a low number of both Tregs and IL-17^+^ cells were correlated with poor disease-specific survival (*p* = 0.007). A low number of Tregs combined with Th17 cells present were also correlated with poor survival (*p* = 0.018). An increased number of IL-17^+^ cells were significantly correlated with the absence of vaso-invasion (*p* = 0.001), smaller tumor size (*p* = 0.030) and less infiltration depth (*p* = 0.021). These results suggest that Tregs and IL-17^+^ cells represent a beneficial immune response, whereas Th17 cells might represent a poor response in cervical adenocarcinoma. This contrasts with the correlations described in squamous cell carcinoma, suggesting that the local immune response in cervical adenocarcinoma contributes differently to tumor growth than in squamous cell carcinoma.

**Electronic supplementary material:**

The online version of this article (doi:10.1007/s00262-015-1678-4) contains supplementary material, which is available to authorized users.

## Introduction

Cervical cancer is the second leading cause of cancer death in young women worldwide [1]. The majority of cervical cancer cases can histologically be divided into squamous cell carcinoma (~75 % of cases), adenocarcinoma and adenosquamous carcinoma (together 20–25 % of cases) [2]. Several studies have reported that the prognosis for patients with cervical adenocarcinoma is worse than for squamous cell carcinoma [3–8], although this is still controversial [9, 10]. Additionally, although the overall incidence of cervical cancer has declined in developed countries as a result of cytological screening programs, the incidence of adenocarcinoma has remained stable or even increased, predominantly in young women [11, 12]. Cervical adenocarcinoma differs from squamous cell carcinoma in growth pattern, molecular background and sensitivity to radio- and chemotherapy [13–15]. However, because of the lower incidence, extensive analyses have been lacking.

Since practically all cases of cervical cancer are caused by a persistent infection with high-risk human papillomavirus (HPV) [16], immunosurveillance plays a critical role. Most cervical HPV infections are cleared in over 90 % of cases within 2 years [17]. In case of tumor progression, the immune response is thought to contribute to tumor development rather than eradication [18]. The type and number of immune cells present in the tumor microenvironment are both crucial for clinical outcome. T helper 1 (Th1) cells are required to overcome intracellular pathogens and can induce or stimulate a tumor-targeting immune response. Th2 cells protect against extracellular pathogens and have been shown to support cervical cancer progression [19], but their role in cancer has not been fully elucidated. Th17 cells are essential to protect against extracellular pathogens and play a dominant role in autoimmune diseases [20, 21]. Their role in cancer is unclear, since they have been shown both to be able to promote and to counteract tumor growth [22]. Regulatory T cells (Tregs) suppress the activity of other T cells [23], which may dampen either a tumor-suppressing or tumor-promoting immune response. In particular, the potentially different role of the immune response in squamous versus adenocarcinoma has not been thoroughly studied, although there are indications for differences between the subtypes [24].

We have shown before that Tregs are more frequently present in cervical squamous cell carcinoma than adenocarcinoma and that these cells, relative to cytotoxic T cells, were correlated with poor survival in a representative cohort of cervical cancer patients, i.e., predominantly squamous cell carcinoma cases (77 %) [25]. In addition, we have recently shown that Th17 cells were correlated with improved survival in a cohort of squamous cell carcinoma patients [26]. Strikingly, interleukin-17 (IL-17) was predominantly expressed by neutrophils and correlated with poor survival in the same cohort [26].

The aim of this study was to determine the number of intraepithelial, stromal and total T cells, Tregs, Th17 and other IL-17^+^ cells. The correlations between the different cell frequencies and patient survival in cervical adenocarcinoma were analyzed. The contrasts with the correlations described in cervical squamous cell carcinoma and other cancer types are discussed.

## Materials and methods

### Patient material

Formalin-fixed, paraffin-embedded (FFPE) cervical adenocarcinoma specimens obtained from all patients who underwent primary surgical treatment for cervical cancer between 1985 and 2005 with sufficient material available for analysis were obtained from the archives of the Department of Pathology, Leiden University Medical Center (*n* = 67). Cervical adenocarcinoma was defined as an invasive epithelial tumor showing glandular differentiation (moderate to highly differentiated) or staining Periodic Acid Schiff Plus and Alcian blue positive and lacking squamous elements (undifferentiated) [27, 28]. None of the patients had received preoperative anticancer therapy, and follow-up data were obtained from patient medical records. Patient and tumor characteristics are listed in Supplementary Table 1. Patient samples were handled according to the medical ethical guidelines described in the Code of Conduct for Proper Secondary Use of Human Tissue of the Dutch Federation of Biomedical Scientific Societies.

### Immunofluorescent staining

Triple immunofluorescent staining was performed on 4-μm-thick FFPE sections. After antigen retrieval using Tris–ethylenediaminetetraacetic acid (EDTA) buffer (10 mM Tris plus 1 mM EDTA pH 9.0), rabbit anti-CD3 (ab828, Abcam, Cambride, UK), mouse IgG1 anti-FoxP3 (ab20034, Abcam) and goat anti-IL-17 (AF-317-NA, R&D Systems, Abingdon, UK) diluted in 1 % w/v bovine serum albumin (BSA) in phosphate-buffered saline (PBS) were incubated at room temperature overnight. Alexa Fluor labeled donkey anti-rabbit-A546 (A10040), donkey anti-mouse-A647 (A31570) and donkey anti-goat-A488 (A11055; all from Invitrogen, Life Technologies, Carlsbad, USA) were incubated at room temperature for 1 h. Slides were mounted using VectaShield mounting medium containing DAPI (Vector Laboratories, Burlingame, USA). For negative controls, the primary antibodies were omitted and substituted with antibodies of the same isotype class with an unknown specificity.

### Microscopic analyses

Immunofluorescent images were acquired with an LSM700 confocal laser scanning microscope equipped with an LCI Plan-Neofluar 25x/0.8 Imm Korr DIC M27 objective (Zeiss, Göttingen, Germany). In the majority of cases, four random images were obtained at a 250× magnification, sampling a total tumor area of generally 1.0 mm^2^, comprising vital areas of both tumor epithelium and stroma. Double or triple positivity of cells as well as the tumor epithelium and stroma area were determined using the overlay tool in the LSM Image Browser software (version 4.2.0.121, Zeiss). The numbers of single, double and triple positive cells were scored in the tumor epithelium and tumor stroma separately using the open source image processing program ImageJ version 1.47 (http://rsb.info.nih.gov/ij). Cells within blood vessels or lumina were not counted.

### Statistical analysis

Statistical analyses were performed using SPSS version 20.0 (IBM Corp., Armonk, USA). Correlations between the number of positive cells and clinicopathological variables were tested using the Spearman’s rank correlation rho (*r*) and Wilcoxon Mann–Whitney tests. Correlations between the number of positive cells and disease-specific or disease-free survival were tested using the Kaplan–Meier and Cox proportional hazards models. For survival analyses, patients were divided in two groups based on the median numbers of positive cells (high and low). All tests were two-sided, and *p* values below 0.05 were considered statistically significant.

## Results

### Infiltrating immune cells

**Fig. 1:**
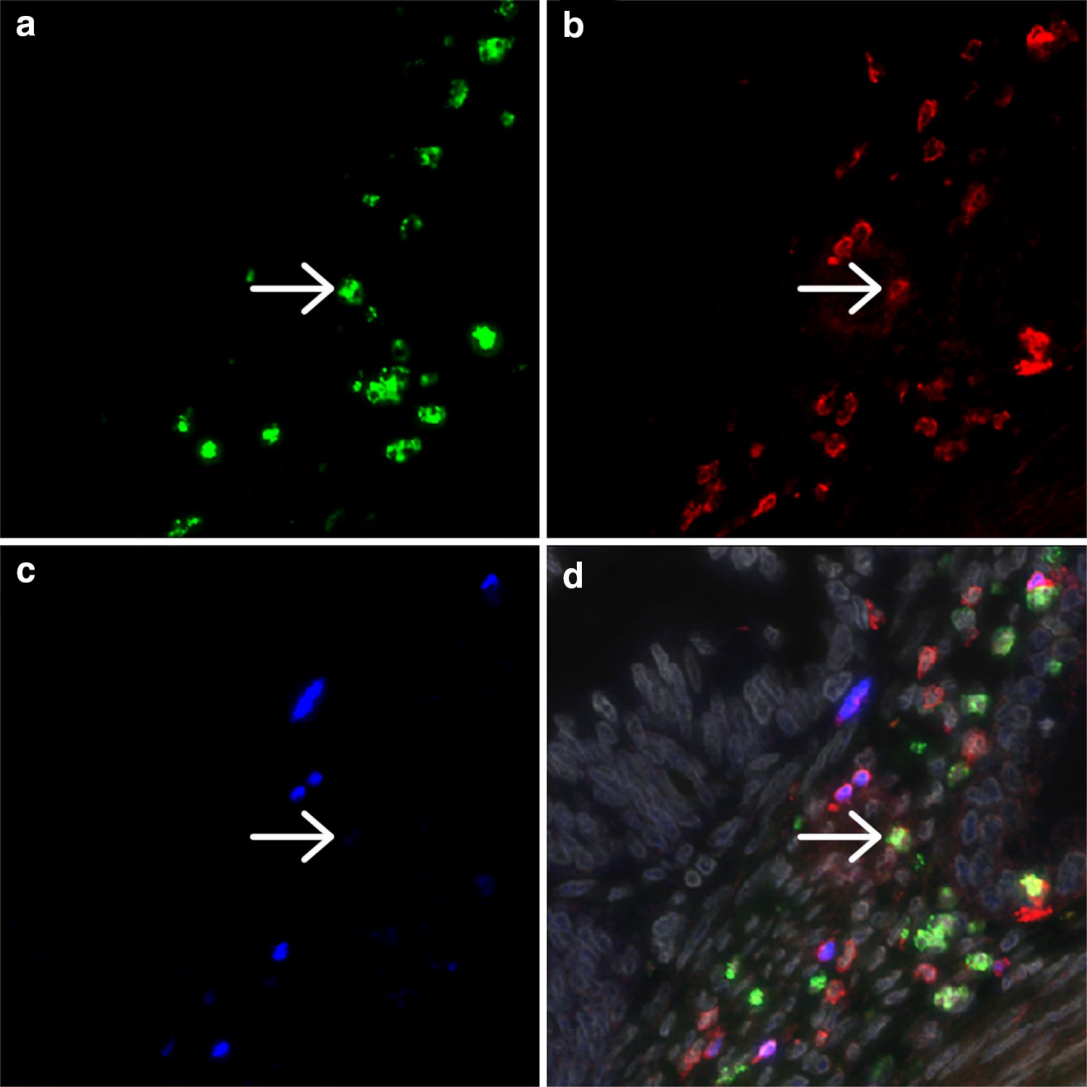
Representative image of a cervical adenocarcinoma specimen stained by triple immunofluorescence for IL-17 (**a**), CD3 (**b**) and FoxP3 (**c**), with the combined staining together with DAPI counterstain (*gray*) shown in (**d**). The *arrow* indicates a cell double positive for IL-17 and CD3. Different CD3/FoxP3 double positive cells are present

The cell density for all cell types analyzed was higher in the tumor stroma than in the epithelium (Fig. 1). Tumor infiltration by CD3^+^ T cells was observed in all samples analyzed, while only a minor population of Th17 cells was observed (Supplementary Fig. 1, Supplementary Table 2). FoxP3^+^ cells were always positive for CD3. Since a single FoxP3^+^ IL-17^+^ cell was only observed in two tumor samples (0.02 % of FoxP3^+^ cells), these cells were not further analyzed. Although approximately three times more CD3^+^ T cells and four times more (non-Th17) IL-17^+^ cells were present in the tumor stroma compared with the epithelium, especially Tregs were more strongly represented in the tumor stroma with on average over ten times higher cell counts.

### Correlation between infiltrating immune cells and survival

**Fig. 2:**
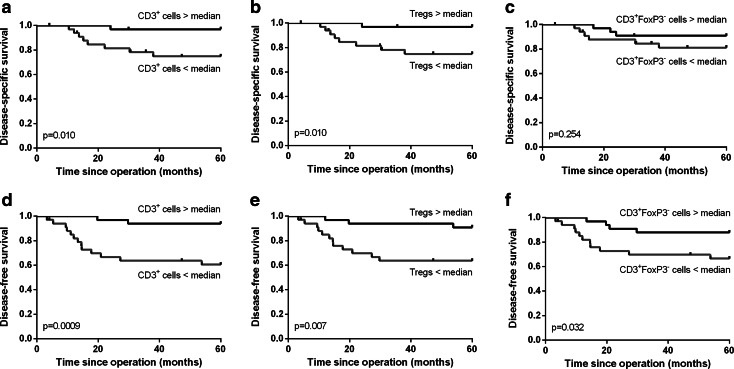
Kaplan–Meier survival curves for a high versus low number of total CD3^+^ T cells (**a**, **d**), CD3^+^FoxP3^+^ Tregs (**b**, **e**) and CD3^+^FoxP3^−^ T cells (**c**, **f**) are shown for disease-specific (**a**–**c**) and disease-free survival (**d**–**f**)

**Fig. 3:**
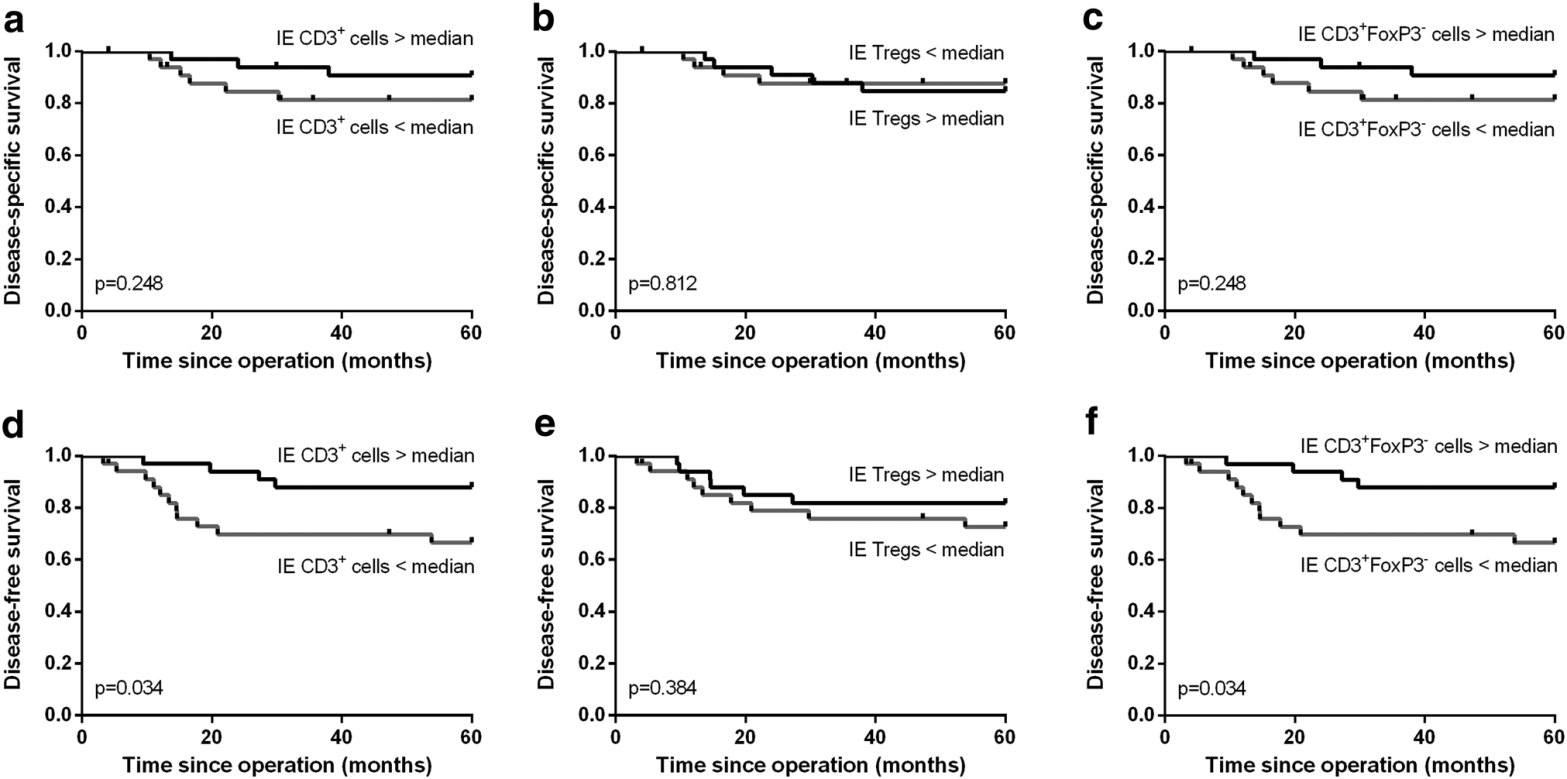
Kaplan–Meier survival curves for a high versus low number of CD3^+^ cells (**a**, **d**), CD3^+^FoxP3^+^ Tregs (**b**, **e**) and CD3^+^FoxP3^−^ T cells (**c**, **f**) infiltrating in the tumor epithelium (IE) are shown for disease-specific (**a**–**c**) and disease-free survival (**d**–**f**)

A high total number of T cells were significantly correlated with improved disease-specific (*p* = 0.010, Fig. 2a) and disease-free survival (*p* = 0.001, Fig. 2d). This was specifically due to a high number of CD3^+^FoxP3^+^ Tregs, since a high number of CD3^+^FoxP3^−^ T cells were less strongly correlated with disease-free survival (*p* = 0.032, Fig. 2f) than a high number of Tregs (*p* = 0.007, Fig. 2e). More importantly, there was no significant correlation between a high number of CD3^+^FoxP3^−^ T cells and disease-specific survival (*p* = 0.254, Fig. 2c), but high Tregs were significantly correlated with improved disease-specific survival (*p* = 0.010, Fig. 2b). Additionally, a high number of total CD3^+^ T cells within the tumor epithelium were significantly correlated with improved disease-free (*p* = 0.034, Fig. 3d) but not with disease-specific survival (*p* = 0.248, Fig. 3a). These correlations were practically similar for the number of CD3^+^FoxP3^−^ T cells (Fig. 3c, f), because the number of FoxP3^+^ cells infiltrating in the tumor epithelium was relatively low. The number of Tregs in the tumor epithelium was not significantly correlated with disease-free or disease-specific survival (Fig. 3b, e). The separate analyses of the correlation between the other cell types present in the tumor epithelium or the tumor stroma compartment and survival were not significant.

**Fig. 4:**
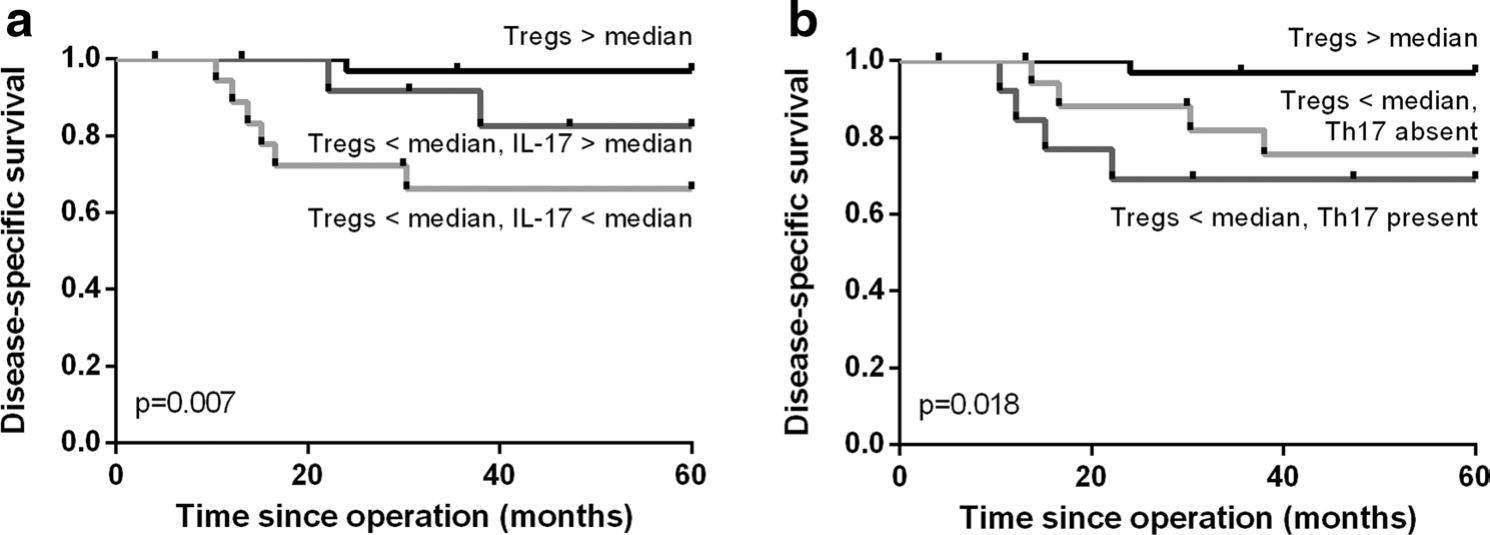
Kaplan–Meier survival curves for disease-specific survival based on a high number of Tregs compared with a low number of Tregs combined with high or low IL-17^+^ cells (**a**) or the absence or the presence of Th17 cells (**b**)

A high number of (non-Th17) IL-17^+^ cells per se were not significantly correlated with survival. However, when combining the Treg and IL-17 scores, patients could be better categorized in groups with poor or improved survival. Compared with a high number of Tregs and a low Treg number but high IL-17^+^ cells, the combination of a low number of both Tregs and IL-17^+^ cells was correlated with worse disease-specific survival (*p* = 0.007, Fig. 4a). Since a high number of Tregs were correlated with favorable prognosis, the number of IL-17^+^ cells did not discriminate between patients with poor or improved survival in this patient group. Having a low number of Tregs and Th17 cells present was correlated with worse survival than high Tregs or low numbers of Tregs and the absence of Th17 cells (*p* = 0.018, Fig. 4b). So despite the generally low numbers of Th17 cells present, their presence still contributed to the effect of the number of Tregs present. Both correlations were also significant for disease-free survival (data not shown).

### Hazard ratio for low Tregs and IL-17^+^ cells infiltration

The hazard ratio for disease-specific survival in case of a low number of Tregs was 9.38 (95 % CI 1.17–75.09, *p* = 0.035, Table 1). This remained significant when correcting for tumor lymph node metastasis (TNM) stage. The hazard ratio for disease-specific survival for tumors with a low number of both Tregs and IL-17^+^ cells was 13.91 (95 % CI 1.67–115.73, *p* = 0.015, Supplementary Table 3) when compared with a high number of Tregs, which also remained significant after correcting for TNM stage. A low number of Tregs combined with the presence of Th17 cells gave a hazard ratio of 12.83 (95 % CI 1.43–114.93, *p* = 0.023, Supplementary Table 4) when compared with a high number of Tregs or low Tregs and absence of Th17 cells, also still significant after correcting for TNM stage.

**Table 1:** Hazard ratio for a low Tregs frequency

Variable	Univariate Cox regression		Multivariate Cox regression	
	Hazard ratio (95 % CI)	*p* value	Hazard ratio (95 % CI)	*p* value
TNM stage	1.606 (1.133–2.276)	0.008	1.643 (1.144–2.361)	0.007
Tregs low	9.384 (1.173–75.093)	0.035	10.131 (1.256–81.719)	0.030

Univariate and multivariate Cox regression analyses for the TNM stage and a low number of Tregs on disease-specific survival are shown. The 95 % confidence interval (95 % CI) of the hazard ratio is indicated within parentheses

### Correlation between IL-17^+^ cells and clinicopathological parameters

**Fig. 5:**
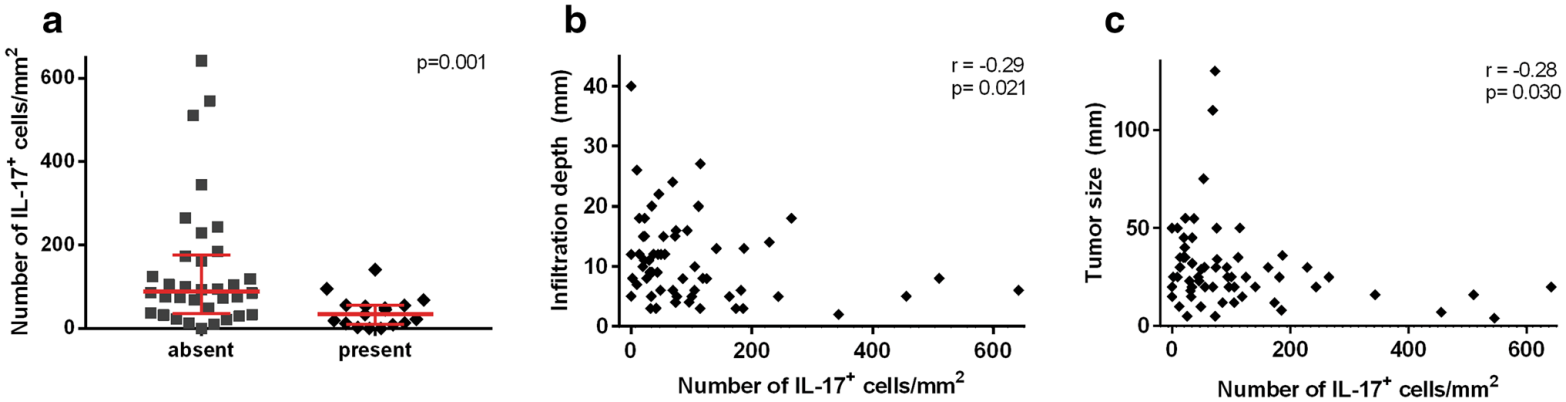
Correlation between the number of total non-Th17 IL-17^+^ cells and vaso-invasion is shown in **a**. The *p* value was calculated using the Wilcoxon Mann–Whitney test. The correlations between the number of non-Th17 IL-17^+^ cells and the continuous variables tumor infiltration depth (**b**) and tumor size (**c**) were calculated using the Spearman’s rank correlation rho test

Finally, we investigated whether the different cell populations were associated with prognostic clinicopathological parameters (lymph node metastasis, tumor size, vaso-invasion and infiltration depth). A high number of IL-17^+^ cells were significantly correlated with the absence of vaso-invasion (*p* = 0.001, Fig. 5a), decreased tumor infiltration depth (*r* = −0.29, *p* = 0.021, Fig. 5b) and decreased tumor size (*r* = −0.28, *p* = 0.030, Fig. 5c). No other significant correlations were found.

## Discussion

The current study showed that a high total number of Tregs were significantly correlated with improved disease-free and disease-specific survival in cervical adenocarcinoma patients. Although tumor-infiltrating immune cells are more frequently present in tumor stroma than in tumor epithelium, especially Tregs were about three times less frequently present in the tumor epithelium than T cells and IL-17^+^ cells. Within the tumor epithelium, a high T cell frequency was significantly correlated with improved disease-free survival. Strikingly, specifically a low total number of both Tregs and IL-17^+^ cells were strongly correlated with poor survival. The IL-17^+^ cells were inversely correlated with vaso-invasion, tumor size and infiltration depth. The number of IL-17^+^ cells could thus further discriminate between patient prognoses after Treg determination. In addition, a low number of Tregs combined with the presence of Th17 cells were correlated with worse prognosis.

The current data suggest that, of the immunological parameters studied, the total number of Tregs is the most important determinant correlated with survival for cervical adenocarcinoma patients. Tregs thus seem to represent a beneficial immune response in cervical adenocarcinoma, which contrasts Tregs correlating with poor survival in cervical squamous cell carcinoma [25, 29]. This corresponds with studies that indicate that cervical adenocarcinoma differs substantially from squamous cell carcinoma [13–15, 24] and suggests that the composition and effect of the tumor-infiltrating immune cells differ per histological tumor subtype. However, a direct correlation between total Tregs and survival in cervical squamous cell carcinoma has not been shown: the significant correlations were specifically found within the tumor epithelium and especially when compared with the number of cytotoxic T cells present. A specific correlation between a high ratio of total T cells or CTL over Tregs and improved survival has recently also been shown in glioblastoma [30]. When we studied the tumor epithelium separately, total T cell frequency was correlated with improved disease-free survival. The latter correlation was irrespective of Tregs, because the intraepithelial T cell frequency predominantly comprised FoxP3^−^ cells. Thus, intraepithelial T cell infiltrate seems to be a general marker for improved survival. These intraepithelial T cells might predominantly be cytotoxic T lymphocytes (CTL). Another partial explanation for the differences found between the histological subtypes might be that the tumors of this cervical adenocarcinoma cohort were generally smaller in size than the squamous cell carcinomas, as was described before [27]. Supporting our data, other studies have also reported correlations between Tregs and poor survival [31–33], indicative of the dampening of an anti-tumor immune response. However, Tregs have also been found to be correlated with improved prognosis in different types of cancer [34–37], suggesting that they may also dampen a tumor-promoting immune response. Indeed, the role of Tregs in cancer is controversial and seems to be context and tumor type dependent [38]. The current data support a predominant role in suppressing tumor growth, favoring inflammation in cervical adenocarcinoma.

IL-17 has, in general, been shown to correlate with poor survival, and Th17 cells with improved survival in cancer [39]. The pro-inflammatory cytokines IL-6 and IL-23, which are implicated in the induction of IL-17 expression [40], have also been shown to be correlated with poor survival in cervical cancer [41]. Since IL-17 has been shown to be generally expressed by granulocytes [26], this suggests that a pro-inflammatory environment may attract granulocytes and other innate myeloid cells favoring tumor growth in cervical squamous cell carcinoma. The correlation between increased IL-17^+^ cells and improved survival especially in case of low Treg frequencies in cervical adenocarcinoma suggests that these cells might rather counteract tumor growth in cervical adenocarcinoma. We have previously shown that IL-17-producing cells represent a heterogenous cell population [26], and we propose that IL-17^+^ cells may predominantly represent tumor-targeting myeloid cells in cervical adenocarcinoma, potentially mast cells and type 1 neutrophils and macrophages. Correspondingly, Chen et al. [42] showed that a high number of infiltrating IL-17^+^ cells were significantly correlated with improved survival in a large cohort of gastric adenocarcinoma patients.

Our results showed that the presence of Th17 cells, specifically when a low number of Tregs were present, was correlated with poor survival. A pro-inflammatory Th17 response, despite the low frequencies, might thus rather be correlated with a tumor-promoting immune response. This does correspond with a study by Yan et al. [43], showing that an increased frequency of circulating Th17 cells is correlated with poor survival in hepatocellular cancer. Also this correlation is in contrast with its role in a beneficial immune response in cervical squamous cell carcinoma [26] as well as other cancer types [39].

These different correlations suggest that the local immune response in cervical adenocarcinoma differs substantially from the immune response in squamous cell carcinoma. This might be related to differences in the molecular constitution of the two cancer types. Cervical adenocarcinoma has recently been shown to contain more frequent *TP53* [44] and *KRAS* mutations and less frequent *PIK3CA* and *PTEN* mutations compared with squamous cell carcinoma [45]. In our patient cohort, we found significantly more frequent somatic *KRAS* mutations in cervical adenocarcinoma, whereas *PIK3CA* mutations were more frequently found in squamous cell carcinoma (manuscript submitted). We have furthermore shown that CXC chemokine receptor 4 (CXCR4), CXCR7 and epidermal growth factor receptor (EGFR) were more frequently expressed in cervical squamous cell carcinoma than adenocarcinoma [46]. Additionally, we showed that HLA-E was overexpressed more frequently in cervical adenocarcinoma than squamous cell carcinoma [27]. High HLA-E expression was significantly correlated with improved disease-free and disease-specific survival in cervical adenocarcinoma, while no correlation was found in squamous cell carcinoma. Cervical adenocarcinoma samples have also been shown to produce higher levels of transforming growth factor-β (TGF-β) than squamous cell carcinoma samples [24]. Since we showed in the present study that Tregs, Th17 cells and other IL-17^+^ cells also show opposed correlations in cervical adenocarcinoma than in squamous cell carcinoma, this suggests that the molecular differences are correlated with a different type of immune response. We speculate that the increased HLA-E and TGF-β expression might cause an effector T cell response to have limited efficacy. Under these circumstances, classically activated myeloid cells such as type 1 neutrophils might be more effective in cervical adenocarcinoma. Infiltration of T cells into the tumor epithelium was correlated with improved survival in both cervical cancer types.

To conclude, our data show that the role of T cells, including Tregs, Th17 cells and other IL-17^+^ cells, is context and tumor type dependent. Tregs and IL-17^+^ cells represented a beneficial immune response correlated with improved survival, while Th17 cells might contribute to tumor progression and poor prognosis in cervical adenocarcinoma. Future research should determine how these cell types are correlated with improved prognosis, what other immune cell types are involved and how this might be used to guide patient prognosis and treatment.

### Electronic supplementary material

Below is the link to the electronic supplementary material.

Supplementary material 1 (PDF 310 kb)

## Abbreviations

BSA: Bovine serum albumin
CI: Confidence interval
CXCR: CXC chemokine receptor
EDTA: Ethylenediaminetetraacetic acid
EGFR: Epidermal growth factor receptor
FFPE: Formalin-fixed, paraffin-embedded
HPV: Human papillomavirus
IL: Interleukin
PBS: Phosphate-buffered saline
TGF-β: Transforming growth factor-β
Th: T helper
TNM: Tumor lymph node metastasis stage
Treg: Regulatory T cell
