# Supplementary material for: FoxP3+ and IL-17+ cells are correlated with improved prognosis in cervical adenocarcinoma
Source: Cancer Immunol Immunother. 2015 Mar 21;64(6):745–53. doi: 10.1007/s00262-015-1678-4 (PMC4456995; doi:10.1007/s00262-015-1678-4)
Supplement: Supplementary file 1 — Supplementary material 1 (PDF 310 kb) [file 262_2015_1678_MOESM1_ESM.pdf]

Cancer Immunology, Immunotherapy (submitted in 2015) – Simone Punt et al.

Supplementary Figures and Tables

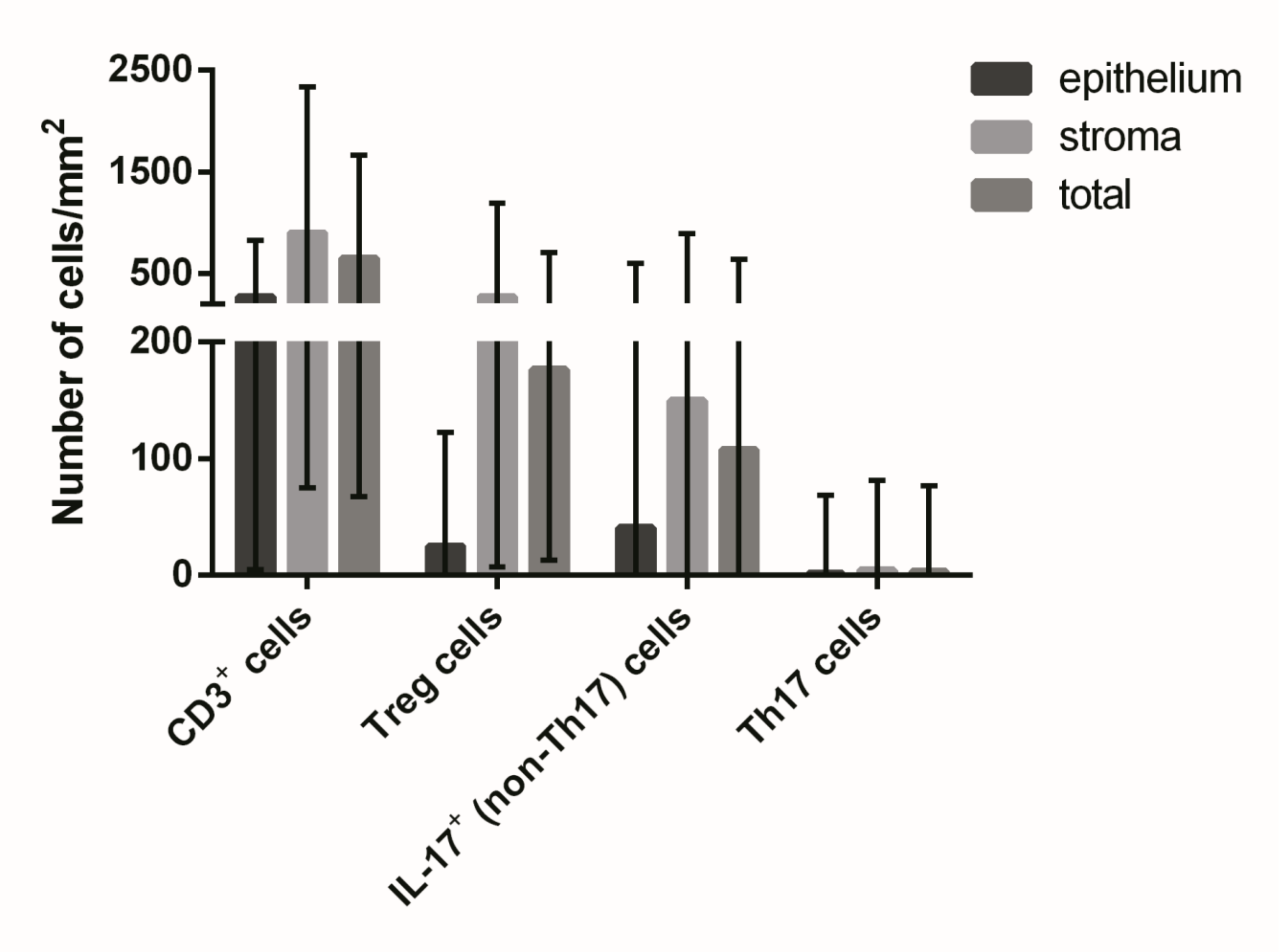

Supplementary Figure 1. Quantification of tumor infiltrating cells

The mean and range of the number of total $CD3^+$ T cells, $FoxP3^+CD3^+$ Tregs, $CD3^-IL\text{-}17^+$ cells and $CD3^+IL\text{-}17^+$ Th17 cells observed infiltrating in the tumor epithelium, tumor stroma and combined total area per mm^2 is shown (n=67).

Supplementary Table 1 Patient clinicopathological characteristics

**Clinicopathological parameter**	**Category**	**N = 67** (%)
**Age**	Median	40
	Range	26-80
**FIGO stage**[a]	IA	2 (3)
	IB	59 (88)
	IIA	6 (9)
**TNM stage**	IAII	4 (6)
	IB1	40 (60)
	IB	4 (6)
	IB2	8 (12)
	IIA	7 (10)
	IIB	1 (1)
	IIIA	1 (1)
	IIIB	2 (3)
**Lymph nodes**	negative	54 (81)
	positive	13 (19)
**Tumor size (mm)**[b]	<40	52 (78)
	≥40	12 (18)
**Vaso-invasion**[b]	Absent	36 (54)
	Present	17 (25)
**Infiltration depth (mm)**[b]	<15	46 (69)
	≥15	17 (25)
**HPV type**	16	26 (39)
	18	24 (36)
	other	17 (25)

[a]FIGO, International Federation of Gynecologists and Obstetricians

[b]Data were not available for all patients.

Supplementary Table 2 Number of T cells, Tregs and IL-17^{+} cells present in cervical adenocarcinoma

	$CD3^{+}$ cells		Treg cells		IL-17^{+} (non-Th17) cells		Th17 cells	
	mean (range)	median	mean (range)	median	mean (range)	median	mean (range)	median
**Epithelium**	268 (5-827)	187	25 (0-123)	17	41 (0-601)	7	2 (0-69)	0
**Stroma**	898 (75-2338)	762	271 (7-1193)	175	150 (0-889)	95	4 (0-82)	0
**Total**	649 (68-1664)	601	176 (13-705)	119	107 (0-642)	69	3 (0-76)	0

The mean, minimum, maximum and median number of total $CD3^{+}$ cells, $FoxP3^{+}CD3^{+}$ Tregs, $CD3^{-}$IL-17^{+} cells and $CD3^{+}$IL-17^{+} Th17 cells observed infiltrating in the tumor epithelium, tumor stroma and combined total area per mm^{2} is indicated.

Supplementary Table 3 Hazard ratio for Tregs in combination with IL-17^{+} cells

**Variable**	**Univariate Cox regression**		**Multivariate Cox regression**	
	Hazard ratio (95% CI)	p value	Hazard ratio (95% CI)	p value
TNM stage	1.606 (1.133-2.276)	0.008	1.545 (1.051-2.273)	0.027
Tregs high	reference		reference	
Tregs low, IL-17 high	5.587 (0.506-61.635)	0.160	7.561 (0.659-86.742)	0.579
Tregs low, IL-17 low	13.906 (1.671-115.730)	0.015	12.261 (1.454-103.383)	0.021

Univariate and multivariate Cox regression analyses for the TNM stage and a low number of Tregs combined with a high or low number of (non-Th17) IL-17^{+} cells versus a high number of Tregs on disease-specific survival are shown.

Supplementary Table 4 Hazard ratio for Tregs in combination with Th17 cells

Variable	Univariate Cox regression		Multivariate Cox regression	
	Hazard ratio (95% CI)	p value	Hazard ratio (95% CI)	p value
TNM stage	1.606 (1.133-2.276)	0.008	1.619 (1.131-2.317)	0.008
Tregs high	reference		reference	
Tregs low, Th17 absent	8.361 (0.934-74.863)	0.058	8.460 (0.942-75.983)	0.057
Tregs low, Th17 present	12.825 (1.431-114.930)	0.023	14.668 (1.603-134.179)	0.017

Univariate and multivariate Cox regression analyses for the TNM stage and a low Tregs number combined with a high or low number of Th17 cells versus a high number of Tregs on disease-specific survival are shown.
